# Clinician’s Guide to Material Selection for All-Ceramics in Modern Digital Dentistry

**DOI:** 10.3390/ma18102235

**Published:** 2025-05-12

**Authors:** Cristiana Cuzic, Mihai Rominu, Alisia Pricop, Horatiu Urechescu, Marius Octavian Pricop, Raul Rotar, Ovidiu Stefan Cuzic, Cosmin Sinescu, Anca Jivanescu

**Affiliations:** 1 Department of Prosthodontics, Faculty of Dental Medicine, University of Medicine and Pharmacy “Victor Babeș”, 300041 Timișoara, Romania; pricop.cristiana@umft.ro (C.C.); rotar.raul@umft.ro (R.R.); jivanescu.anca@umft.ro (A.J.); 2 TADERP Research Center—Advanced and Digital Techniques for Endodontic, Restorative and Prosthetic Treatment, University of Medicine and Pharmacy “Victor Babeș”, Revolutiei Ave. 1989, No. 9, 300041 Timișoara, Romania; 3 Research Center in Dental Medicine Using Conventional and Alternative Technologies, School of Dental Medicine, University of Medicine and Pharmacy “Victor Babeș”, 300070 Timișoara, Romania; rominu.mihai@umft.ro (M.R.); pricop.marius@umft.ro (M.O.P.); sinescu.cosmin@umft.ro (C.S.); 4 Department of Prosthesis Technology and Dental Materials, Faculty of Dentistry, University of Medicine and Pharmacy “Victor Babeș”, 300041 Timișoara, Romania; 5 Department of Oral and Maxillo-Facial Surgery, Faculty of Dental Medicine, University of Medicine and Pharmacy “Victor Babeș”, 300041 Timișoara, Romania; 6 Department of Hydrotechnical Engineering, Faculty of Civil Engineering, Politehnica University Timișoara, 300006 Timișoara, Romania; ovidiu.cuzic@student.upt.ro

**Keywords:** all-ceramic restorations, modern dentistry, computer-aided design, dental ceramics, adhesion, resin cements

## Abstract

All-ceramic restorations are the foundation of modern restorative aesthetic dentistry. The industry for dental materials now provides a large selection of biomaterials with a range of constantly improving qualities. Although this is undoubtedly advantageous, the vast array of materials may confuse even experienced dentists. Even if recently the demand of digital dentistry in daily dental practice has significantly increased, due to a lack of understanding concerning cementation techniques, which are different for each type of ceramic used, dentists are continuing to be hesitant to utilise these various CAD/CAM materials. This study analysed 58 articles from 2008 to 2025, focusing on narrative, comprehensive, and systematic reviews and in vitro studies on dental dentistry materials. English articles were included, but non-English articles and case reports were excluded. The analysis included articles from all journal categories, ensuring adherence to inclusion and exclusion criteria. The aim of the research is to assess material classifications and properties that guide practices concerning the adhesive cementation of all-ceramic restorations. In order to provide a clear overview of the composition, characteristics, clinical considerations, and current trends of contemporary dental materials, as well as some recommendations for future research in this area that would be relevant to dentists and the scientific community, the authors of the paper were guided by this structure when writing the article content. The key is to ensure the aesthetics, resistance, and long-term clinical success of the treatment plan by providing dental professionals with clear, accurate information and instructions about resin-luting materials and indirect restoration materials.

## 1. Introduction

Digital dentistry is undergoing a significant transition due to advancements in CAD/CAM technology and innovative aesthetic materials. This has significantly impacted clinical procedures, workflow, patients’ desires, and treatment plan options. Modern treatment options are “minimally invasive” and focus on “preserving dental tissue” [1]. Over the last 20 years, dental ceramics and processing technologies have advanced, leading to a shift towards monolithic restorations [2]. Monolithic prostheses support clinicians with managing the fracture of the low-strength veneering layer in multilayered restorations. However, they may also face other clinical issues like wear of the antagonist dentition and matching the natural dentition’s aesthetic characteristics. Successful restorations require an understanding of material dynamics [3].

The selection of restorative materials and luting cements in modern therapeutic settings has become increasingly complex due to the introduction of new cement advancements. Clinicians face a complex variety of resin cements, each with distinct polymerisation mechanisms (light-cured, self-cured, or dual-cured), differing in adhesive strengths, handling characteristics, and indications. Factors such as the translucency and thickness of the ceramic restoration, the accessibility of the preparation for light-curing, and the need for immediate versus extended working time all contribute to the difficulty in choosing the optimal cement for a given clinical situation. This necessitates management in various clinical scenarios that may not be compatible with certain prosthetic materials [4]. To achieve the best results, practitioners must consider the properties and applicability of each substance, including bond longevity, aesthetics, and biocompatibility [5].

Due to their microstructure and chemical composition, ceramic materials may be divided into two major groups: oxide-based ceramics like alumina and zirconia and predominantly silica-based with fillers like feldspathic, leucite, or lithium disilicate [6]. The superior aesthetics, biocompatibility, and functional advantages of all-ceramic restorations have established them as the preferred treatment option over the past decade for a wider array of therapeutic applications, including crowns, partial dentures, and implants in anterior regions [7].

The enduring success of a restoration is reflected by its longevity, which is achieved through the selection of a luting agent and cementation method [8]. It is necessary to enhance the ceramic surface in order to produce suitable bonding with the luting agent. Examples of mechanical treatments include air abrasion/sand blasting, diamond stone burs, sandpaper discs, and laser therapy [9,10]. In contrast, excessive surface roughening should be avoided as it may lead to crack initiation and propagation inside the ceramic, ultimately ending with the fracture of the ceramic restoration [11]. Chemical alterations to the ceramic surface may be achieved by etching and improving the mechanical retention of the adhesive or by adjusting the surface’s affinity for adhesive compounds [12,13].

A durable and stable composite resin adhesive bond requires both a correctly etched prosthetic restoration on one side and the tooth surface on the other. Hydrofluoric acid etching modifies the surface morphology of glass matrix ceramic restorations [14], resulting in highly effective micro-retentions that allow the restoration to be reliably bonded using composite resin-luting materials. Additionally, etching allows for the shaping of an intaglio surface with a thin but presumably considerable graded layer and may repair surface damage in particle-filled glasses caused by adjustments or sandblasting [6]. Chlorhexidine can be applied after acid etching to minimise bond interface degradation in etch-and-rinse dentin adhesives [15]. It preserves collagen integrity and inhibits matrix metalloproteinase, reducing degradation. However, it is not recommended for self-etch or self-adhesive resin cements, as it may negatively affect their bond integrity when bonded to dentin [16].

Studies have shown that chemical conditioning treatments, such as silanization, improve the adhesion of composite resin bonds to ceramics [17]. The silanization process chemically bonds the silica of the dental ceramic with the acrylic group of the composite resin. A suggested method involves a combination of mechanical and chemical conditioning techniques designed to improve the bonding strength of adhesive resins to ceramics [12,18]. Performing more pressure during the bonding process may be advantageous. This approach can lower water absorption, hence reducing water penetration into the underlying dentin as well as strengthening the quality of the adhesive contact [19].

Dental cement should possess the mechanical, biological, physical, and handling qualities required to guarantee restorative retention to the substrate [17]. These characteristics include biocompatibility with pulp and adjacent soft tissues, effective physical characteristics like low solubility, extended working duration, brief setting time, radiopacity, optimal film thickness [20], elevated shear, tensile and compressive strength, excellent adhesion to both the substrate and the restorative material, easy mixing and manipulation, and facile removal of surplus cement after the cementation process [21].

The success of minimally invasive treatment alternatives by adapting the cementation procedure of CAD/CAM materials represents a demanding issue, leading to the fundamental topic of this manuscript. The aim was to provide a comprehensive overview of the composition, characteristics, and contemporary advancements of modern dentistry while offering potential research directions in this domain that would be advantageous for dental clinicians and researchers. To achieve this aim, the specific objectives of this literature review are as follows: first, to examine the current status of research concerning the composition, and contemporary progress in all-ceramic materials within modern dentistry; second, to synthesise the mechanical and optical properties of all-ceramic restorative materials; third, to present an overview of current cementation techniques and their relevance to diverse all-ceramic materials; fourth, to identify potential directions for future research that could impact both dental clinicians and researchers; and finally, to offer a practical guide to assist clinicians in the selection of all-ceramic materials based on their properties and clinical indications.

## 2. Materials and Methods

This literature review evaluated and compared studies based on digital dentistry materials and their bond. The methodology for the buildup of this article is outlined as follows: Specific research was identified through searches in the Google Scholar, PubMed/Medline, and Web of Science databases, focusing on publications from 2008 to 2025. English articles were incorporated. The following keywords and combinations were utilised: “all-ceramic restorations”, “modern dentistry”, “computer-aided design”, “dental ceramics”, “adhesion”, and “resin cements”. This study concentrated on narrative, comprehensive, and systematic reviews and in vitro studies that detail materials used in digital dentistry. Nevertheless, articles in other languages than English and case reports were excluded from the analysis. Articles from all journal categories were included, contingent upon their adherence to the established inclusion and exclusion criteria. A total of 58 were selected. In addition, supplementary references are included to support accurate findings. The article is divided into two parts: (1) classification, properties, and applicability of dental ceramic materials; (2) adhesive materials for all-ceramic dentistry.

## 3. An Updated Overview of Dental Ceramics

### 3.1. Classification of Dental Ceramics According to Material Composition

A classification system for ceramic materials in dentistry offers various purposes, including communication and the educational process. The ideal classification system should include clinically relevant information about the intended use of the material, its location area (anterior vs. posterior), and the most suitable type of restoration (partial vs. full, short-term vs. long-term). Several classifying methods have been suggested, including those based on clinical indications, composition, etching conditions, processing techniques, microstructure, translucency, and fracture resistance [22,23]. Nonetheless, these categories sometimes reflect a lack of definition and clarity, complicating the use of new restoration materials.

A beneficial first approach would be to categorise ceramics according to their chemical composition, since a technical and clinical comprehension of this composition is needed for optimal results. Regrettably, so far, initiatives have shown a lack of specificity and feasibility [6].

Aesthetic dentistry has been founded on ceramics for more than a century. Ceramics were first used mostly for anterior teeth as high-fusing porcelain jacket crowns, partial coverage crowns, or complete denture teeth in their naturally occurring feldspathic form. Since John McLean first developed aluminous porcelain in the mid-1960s, continuous improvements in strength, appearance, and manufacturing procedures have resulted in a wide range of different products for dentists to choose from [24].

Since there are so many alternatives available and new products are being delivered rapidly, clinicians today have to decide when selecting ceramic restoration materials for certain indications. The selection is generally based on a comprehensive understanding of the material properties. It is increasingly influenced by elements such as in vitro strength evaluations, translucency, manufacturing techniques, the preferences of dental laboratory technicians, and advertising statements [6].

Both amorphous and crystalline materials may be identified as ceramics [25,26]. Consequently, ceramics may be generically divided as crystalline and non-crystalline (amorphous solids or glasses) ceramics. The mechanical and aesthetic properties of dental ceramics are mostly determined by the type and proportion of crystalline phase present. Increased glassy phases optimise ceramic translucency; yet they simultaneously compromise structural integrity by reducing crack propagation resistance. Still, the mechanical characteristics will improve with a higher crystalline phase, which will affect the aesthetics [23,25]. Conventional or feldspathic ceramics are often non-crystalline elements. A combination of their fragility and brittleness, these porcelains can fracture even under minimal stress. The ongoing development of crystalline porcelains using adequate fillers such as alumina, zirconia, and hydroxyapatite is attributable to advances in the processing technology of the dental ceramics [25,27,28].

Glass matrix and crystalline structures are fundamental elements of dental ceramics. The most common crystalline minerals include feldspar, quartz, alumina, and perhaps kaolin as a glass matrix (Table 1) [23,25,26].

Based on their glass content, Kelly and Benetti [29] created a widely used classification system that separates ceramic materials into three groups: (1) primarily glassy materials; (2) particle-filled glasses; and (3) polycrystalline ceramics free of glass. The lack of precision in attempting to identify the amount of glass phase required for the ceramic to be categorised through this approach of glass content classification as either predominantly glassy or particle-filled glasses may cause confusion for the clinician. Furthermore, a clear correlation between the quantity of glass and the strength and aesthetics of the ceramic restoration has been suggested. In light of this, glassy ceramics are often precise aesthetically, but polycrystalline ceramics are far less so and are only appropriate for use as a framework for prosthodontic restorations. In a way, it suggests that ceramics composition and its indications are correlated [30]. The concept has been challenged by the latest advances in polycrystalline ceramic microstructure; as stronger but more opaque glass ceramics and more transparent zirconia have become available, aesthetics is becoming less of a concern. Consequently, translucent and aesthetic zirconia may now be used for full-contour restorations and as a framework for veneered core multi-unit bridges, as previously recommended [31,32,33]. 

**Figure 1:**
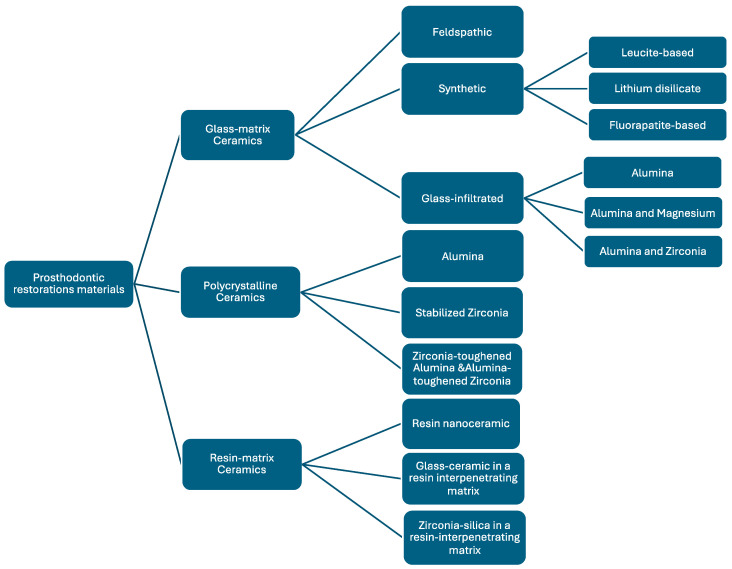
Overview of classification system for all-ceramic and ceramic-like materials.

Gracis et al. (2015) [6] introduced a new classification for dental ceramics, emphasising the wide variety of ceramic systems now accessible (Figure 1).

This classification was determined by the composition concerning the presence or absence of the glass phase and organic matrix: polycrystalline ceramics require inorganic and non-metallic materials without a glass phase, whereas glass matrix ceramics must include non-metallic inorganic ceramic materials that contain a glass phase in their composition. Additionally, the resin matrix ceramic groups must have a polymer matrix containing mostly inorganic refractory compounds.

**Figure 2:**
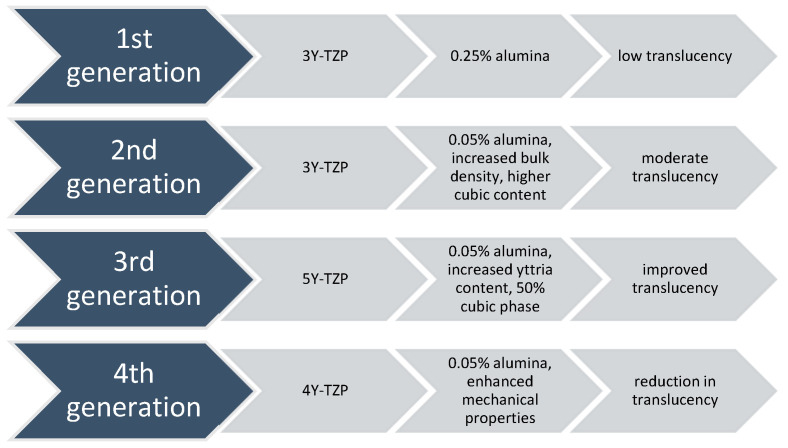
The composition and translucency levels of zirconia ceramic generations [49].

The classification is described in detail and contains contemporary understanding and refined categories incorporating advancements in material science:

1. Glass matrix ceramics are a class of inorganic, non-metallic materials created through specifically crystallising glasses using a variety of processing methods [34]. These ceramics are divided into three subgroups: glass-infiltrated, synthetic, and feldspathic ceramics [35]. Their ability to replicate dental tissues and their biocompatibility and chemical endurance in the oral environment, along with their favourable mechanical qualities, make them popular for use in aesthetic restorations for anterior teeth [36].
  1.1. Felspathic ceramic (e.g., IPS Classic by Ivoclar Vivadent AG, Schaan, Liechtenstein; Vitadur, Vita VMK 68, Vitablocs Mark II, and specific Vitablocs from VITA Zahnfabrik, Bad Säckingen, Germany). These materials are still used as veneers on ceramic and metal alloy frames [37] and as an aesthetic material bonded to dental structures [6,23,38]. Dental ceramics are made of feldspar, quartz, and kaolin, with quartz [39] being the primary component for restoration translucency. Alumina is added to increase strength, while kaolin, a hydrated aluminium silicate, binds ceramic particles together [14]. VITABLOCS from VITA Zahnfabrik, Bad Säckingen, Germany, a feldspar-based CAD/CAM ceramic, is widely used for its ability to replicate natural tooth colours. VITA has introduced new generations, such as TriLuxe (2003) and TriLuxe forte (2007), which have three and four layers with varying shade intensities, making them indicated for partial or full coverage crowns and veneers [40]. VITABLOCS RealLife (2010) VITA Zahnfabrik, Bad Säckingen, Germany enhances the shade gradient with multichromatic feldspar ceramic. VITA Mark II, after hydrofluoric acid surface etching, exhibits micropores and channels with irregular ceramic particles, making it suitable for composite luting cement [41].
  1.2. Synthetic ceramics are popular due to their chemical stability, biocompatibility, translucency, and mechanical strength, making them ideal for non-retentive bonded restorations [36]. Examples include lithium disilicate, leucite-reinforced, fluorapatite-based ceramics, and zirconia-reinforced lithium silicate.

- Leucite-based ceramic (e.g., IPS Empress Esthetic, Ivoclar Vivadent AG, Schaan, Liechtenstein; IPS Empress CAD Ivoclar Vivadent AG, Schaan, Liechtenstein; OPC 3G Kerr Dental (Envista Corporation), Brea, CA, USA) made by processing two basic glasses has a 45% crystalline content of leucite and is fired at 1200 °C, pressed in moulds, and stabilised in cubic form. Its application is limited to veneers, onlays, inlays, and single crowns [42].
- Lithium disilicate ceramic and derivates (e.g., IPS e.max CAD, IPS e.max Press from Ivoclar Vivadent AG, Schaan, Liechtenstein; Obsidian from Glidewell Laboratories, Newport Beach, CA, USA; VITA Suprinity from VITA Zahnfabrik, Bad Säckingen, Germany; Celtra Duo, Dentsply Sirona, Charlotte, NC, USA). The IPS Empress 2 from Ivoclar Vivadent AG, Schaan, Liechtenstein, introduced in 1998, is a ceramic material derived from the crystallisation of a SiO_2_-Li_2_O precursor glass into lithium disilicate and smaller amounts of lithium metasilicate [42]. These crystals make up about 70% of the glass ceramic volume, with a thickly interlocked microstructure contributing to its strength. These materials show improved mechanical characteristics that make them suitable for use in crowns in the anterior and posterior area, inlays, onlays, monolithic infrastructures, and anterior three-unit restorations. Its layered crystals and interlocked microstructure contribute to its strength [43].
- Fluorapatite-based ceramic (e.g., IPS e.max Ceram, ZirPress, Ivoclar Vivadent AG, Schaan, Liechtenstein) as a glass ceramic material with fluorapatite crystals for highly aesthetic qualities is suitable for veneers and glazing lithium disilicate frameworks [37].
- Zirconia-reinforced lithium disilicate (e.g., Celtra Duo, Dentsply Sirona, Charlotte, NC, USA; Vita Suprinity, VITA Zahnfabrik, Bad Säckingen, Germany). Adding up to 10% zirconium oxide to precursor glass has been used since 2013 to reinforce synthetic glass ceramics. These blocks are either partly crystallised, which need further heat treatment, or totally crystallised [44]. These materials exhibit superior aesthetic properties, translucency, opalescence, and fluorescence, making their indications [14] comparable to those of lithium disilicate glass ceramics due to their similarity.
  1.3. Glass-infiltrated ceramic (e.g., In-Ceram Alumina, VITA Zahnfabrik, Bad Säckingen, Germany); alumina and magnesium (e.g., In-Ceram Spinell, VITA Zahnfabrik, Bad Säckingen, Germany); alumina and zirconia (e.g., In-Ceram Zirconia, VITA Zahnfabrik, Bad Säckingen, Germany). These dental ceramics are ceramic glass composites with two interpenetrating phases, with aesthetic and strength properties determined by porous core chemical composition [6]. The opaque aspect requires the application of a porcelain veneer to enhance aesthetics. The use of this class of materials decreases as zirconia and lithium disilicate have become more common, particularly for CAD/CAM manufacturing [14].

2. Polycrystalline ceramics are restorative materials with a microstructure consisting of tightly packed crystalline grains, providing superior mechanical properties like increased strength and fracture toughness. The absence of a glassy phase results in distinct poor translucency and aesthetic qualities [45]. Researchers are exploring modifications to enhance properties, balancing strength with aesthetics for optimal clinical performance.
  2.1. Alumina (e.g., Procera AllCeram and Nobel Biocare, Göteborg, Sweden; In-Ceram AL, VITA Zahnfabrik, Bad Säckingen, Germany). Al_2_O_3_, the material’s primary component, has a 99.5% purity level. It was first introduced as the primary component for CAD/CAM manufacturing by Nobel Biocare, Göteborg, Sweden in the middle of the 1990s. Although it is a highly strong and resistant material, its high elastic modulus has made it more susceptible to fractures among all dental ceramics. This vulnerability to core fracture and the development of materials with improved mechanical properties—such as stabilised zirconia’s transformation toughening properties—have led to a decrease in the use of alumina [22,33,46].
  2.2. Stabilised zirconia (e.g., NobelProcera Zirconia, Nobel Biocare, Göteborg, Sweden; Lava/Lava Plus, 3M ESPE, (3M Oral Care), St. Paul, MN, USA; In-Ceram YZ, VITA Zahnfabrik, Bad Säckingen, Germany; Zirkon, DCS Dental AG, Buchs, Switzerland; Katana Zirconia ML, Kuraray Noritake Dental Inc., Tokyo, Japan; Cercon ht, Dentsply Sirona, Charlotte, NC, USA; Prettau Zirconia, Zirkonzahn GmbH, Gais, Italy; IPS e.max ZirCAD, Ivoclar Vivadent AG, Schaan, Liechtenstein; Zenostar, Wieland Dental (Ivoclar Vivadent AG after acquisition), Pforzheim, Germany). Zirconia (ZrO_2_) is a common material for dental restorations because of its excellent mechanical and aesthetic qualities, particularly transformation toughening. Modern chairside milling and rapid-sintering technologies have contributed to the efficiency of the manufacturing process [47]. However, zirconia’s opaque white colour can make it look less desired, so it is often veneered with porcelain to improve aesthetics [48]. Zirconia is a crystalline substance including three main phases: monoclinic, tetragonal, and cubic [49,50]. As it cools, it undergoes a transformation from cubic to monoclinic, leading to a 5% volumetric expansion [51]. This can cause fractures and make it brittle, making it unsuitable for biomedical, structural, or functional applications. Metal oxides like yttrium oxide stabilise the tetragonal phase, with Y_2_O_3_ being the most commonly used. Transformation toughening enhances the material’s physical properties, providing high flexural strength and fracture toughness [52]. However, it still has drawbacks like translucency level, radiopacity, and limited light transmission [53]. 3Y-TZP, a traditional zirconia dental material with 0.25–0.5 wt.% alumina, has been used for over 25 years due to its high mechanical properties and biocompatibility, making it suitable for core materials and multi-unit restorations [54]. Nevertheless, its high opacity limits its aesthetic considerations. In 2013, improved versions were introduced by reducing alumina content [47] and enhancing translucency through high-temperature sintering. The development of highly translucent 3Y-TZP with less than 0.05% alumina and the introduction of 5Y-PSZ, which contains 5 mol% yttria and approximately 50% cubic phase [55], further improved translucency, allowing for monolithic zirconia restorations in anterior teeth [50]. Nonetheless, increasing the cubic phase content can negatively impact mechanical properties like flexural strength and fracture toughness. Efforts to optimise material properties for monolithic zirconia restorations led to the introduction of 4Y-PSZ in 2017, which has a reduced yttria content (4 mol%) compared to 5Y-PSZ, resulting in improved mechanical properties but compromised optical characteristics. 4Y-PSZ is suitable for both anterior and posterior crowns and short-span fixed partial dentures [55], while 5Y-PSZ is mainly for anterior crowns [56]. Higher yttria content enhances optical properties but reduces mechanical strength [57]. Among yttria-stabilised zirconia, 3Y-TZP has the highest mechanical strength but is opaque, limiting its use to non-aesthetic areas. 5Y-PSZ offers better aesthetic outcomes but lower strength, making 4Y-PSZ a balanced option. Increasing the cubic phase in zirconia improves translucency but decreases strength (Figure 2) [58].
  Overall, while third-generation zirconia excels in optical properties, fourth-generation zirconia offers better restoration longevity [59]. The introduction of polychromatic or colour-gradient blocks, which replicate natural tooth colour transitions, combines high strength and enhanced translucency, with high-strength zirconia in the dentin region and high translucency in the incisal or occlusal areas for improved aesthetics. Dental zirconia is now classified into three types: monochromatic zirconia ceramics with uniform composition, polychromatic multilayered zirconia ceramics, and polychromatic hybrid-structured multilayered zirconia ceramics [60].
  2.3. Zirconia-toughened alumina (ZTA) and alumina-toughened zirconia (ATZ) are composite materials where the ratio of zirconia and alumina can be adjusted during manufacturing to achieve specific properties. The quantity of zirconia or alumina in the composite may be adjusted and modified according to customer preferences or production techniques to improve resistance to low-temperature degradation [61]. Notably, the clinician should distinguish between ZTA, which has an alumina matrix with zirconia particles, and ATZ, which has a zirconia matrix with alumina particles, as this significantly influences the resulting material properties. In comparison to Y-TZP, these composite materials claim benefits such as resistance to low-temperature deterioration, superior strength and fracture toughness, and cyclic fatigue strength exceeding twice that of Y-TZP [62,63], while the actual performance is highly dependent on the precise composition and processing of the composite.

3. Resin matrix ceramics are hybrid materials composed of organic polymers and inorganic fillers, typically ground ceramics [64]. They have unique properties due to the interpenetrating nature of organic and inorganic elements. The inorganic component makes up over 60% of the weight, while the organic component consists of a polymer matrix [42]. In contrast to traditional ceramics, the aim of the development of these materials is to more closely replicate the dentin’s elastic modulus. At the same time, the goal was to produce a material that was easier to refine and mill than glass matrix ceramics, making composite resin easier to use for repair or modification [6]. According to Bajraktarova-Valjakova et al. [14], this class of ceramics includes two types of materials: dispersed-phase materials and polymer-infiltrated ceramics. Dispersed-phase materials, first introduced in conservative dentistry in the 2000s, are made from composite resins inserted in a plastic phase. Two composites, nanoceramic resins (NCR) and zirconia–silica, differ in the size of their ground ceramic particles. NCR has nanometric ceramic particles and micrometre-sized zirconia–silica components. Polymerisation is carried out at high temperatures [65]. NCR’s elastic properties make it convenient for intraoral milling and repair with less pulpal damage risk, emphasising clinical performance [66].
  3.1. Resin nanoceramic (e.g., Lava Ultimate, 3M ESPE (3M Oral Care), St. Paul, MN, USA) consisting of nanoscale ceramic particles dispersed within a resin matrix;
  3.2. Resin-infiltrated glass ceramic (e.g., Enamic, VITA Zahnfabrik, Bad Säckingen, Germany) where a glass ceramic network is infiltrated with a polymer;
  3.3. Resin-infiltrated zirconia–silica nanoceramics (e.g., Paradigm MZ100 Blocks, 3M ESPE (3M Oral Care), St. Paul, MN, USA), which combine zirconia–silica nanoparticles within a resin matrix.

**Figure 3:**
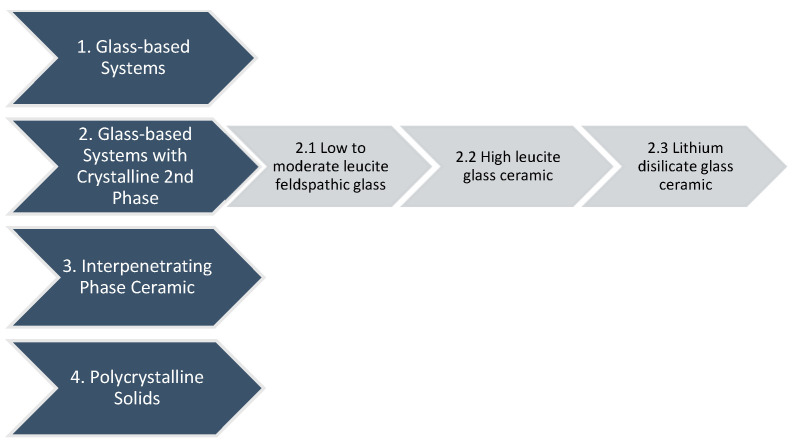
The classification of dental ceramics based on their microstructure [67].

According to the glass-to-crystalline proportion of the ceramic materials, McLaren E.A. and Giordano R. created a new classification based on their microstructure [67]. Materials can have a variety of microstructures, but they can be organised into four groups based on composition (Figure 3): glass-based systems (mostly silica) with and without fillers, generally crystalline (often leucite or even another high-fusing glass), crystalline-based systems with glass fillers (mainly alumina), and polycrystalline solids (alumina and zirconia).

First Category: Glass-based systems

The materials used in the manufacturing of glass-based systems primarily consist of silicon dioxide, referred to as silica or quartz, together with different quantities of alumina (aluminium oxide, Al_2_O_3_). Feldspars are aluminium silicates that occur naturally and vary in potassium and sodium concentration. Feldspars are modified in various ways to produce glasses used in clinical dentistry. Synthetic aluminosilicate glasses are also manufactured for dental ceramics [67].

Second Category: Glass-based systems with a crystalline second phase, porcelain

This category of materials contains a variety of glass–crystalline ratios and crystal types and may be divided into three distinct groups. The composition of the glass is similar to that of Category 1’s pure glass. The difference is that various quantities of multiple crystals have either grown in the glass matrix or been inserted into it.

The three basic crystal types nowadays are leucite, lithium disilicate, and fluorapatite. Leucite is formed in dental porcelain by increasing the potassium oxide component of aluminosilicate glass. Lithium oxide is incorporated into aluminosilicate glass to generate lithium disilicate crystals. Furthermore, it serves as a flux, lowering the melting point of the substance. The survival statistics of this material are comparable to those of metal–ceramic materials [28,68]. The benefit of a prefabricated block is that there is no remaining porosity in the final core, which might represent a flaw and lead to the prosthodontic restoration failing [67].

2.1. Subcategory: Low to moderate leucite-containing feldspathic glass

Such materials frequently appear as “feldspathic porcelains,” although they belong to distinct categories and possess glass comparable to feldspathic types. The strength of the material may be increased by leucite’s ability to affect the coefficient of thermal expansion (CTE) and stop fractures from propagating. The amount of leucite in the glass may be adjusted based on the kind of core and the required CTE [67].

2.2. Subcategory: High leucite-containing (approximately 50%) glass

Homogeneous glass, with its uniform composition, may not possess the desired mechanical and physical properties for certain applications. Secondary heat treatment can nucleate and grow crystals within the glass matrix, providing additional strength and toughness. This process results in glass ceramics, which have superior properties compared to traditional glasses. The interaction of crystals and glass matrix, as well as the size and quantity of crystals, affects their properties. The addition of a second phase, which causes dispersion strengthening, enhances strength and fracture resistance by preventing crack propagation and halting growth [67].

2.3. Subcategory: Lithium disilicate glass ceramic

The flexural strength of a restoration was improved by increasing the crystal concentration to 70% and decreasing its size. The glass matrix is made of lithium silicate and layered with sub-micron orthophosphate crystals. The restoration’s final design and colour can be achieved by adding a porcelain veneer with fluorapatite crystals [69,70,71]. Lithium disilicate crystals have a low refractive index, reflected in their translucency, making them suitable for full crown restorations or aesthetic applications. They can be veneered with specialised porcelain, achieving the desired form and colour. Fluorapatite crystals within an aluminosilicate glass create a glassy phase, making the veneering material and lithium disilicate compound susceptible to etching. Initial clinical results for single restorations are encouraging, especially when adhesively cemented [72].

Third Category: Interpenetrating phase ceramic

VITA In-Ceram, VITA Zahnfabrik, Bad Säckingen, Germany, an all-ceramic restorative material category, was introduced in 1988. It offers a variety of veneers, inlays, onlays, crowns, and bridges. The most transparent material is VITA In-Ceram SPINELL, with high strength for anterior crowns. VITA In-Ceram ALUMINA and VITA In-Ceram ZIRCONIA are used for three-unit posterior bridges [73]. VITA In-Ceram is a porous ceramic used in dental restorations with two interconnected phases. It requires a complex path through alternating layers to fracture. The material is infiltrated with lanthanum aluminosilicate glass, creating a dense interpenetrating composite. This technique, developed as an alternative to metal–ceramic restorations, has shown excellent clinical outcomes [74]. The system contains a sintered crystalline matrix composed of a high-modulus material that constitutes 85% of the volume and includes a junction of crystalline phase particles. This is very different from glasses or glass ceramic materials, which consist of a glass matrix without any particle junctions (crystals) and may or may not contain a crystalline filler. The ceramic matrix can be produced by slip casting or by milling a pre-sintered block [75,76]. The application of VITA In-Ceram ALUMINA, VITA Zahnfabrik, Bad Säckingen, Germany, for single full-coverage crowns throughout the dental arch is supported by various supplementary clinical studies depending upon the quality of the abutment. VITA In-Ceram ZIRCONIA, VITA Zahnfabrik, Bad Säckingen, Germany, should only be applied to molars since it has extremely high opacity, which is inappropriate for anterior aesthetics [77]. Due to its superior translucency (Table 2), VITA In-Ceram SPINELL, VITA Zahnfabrik, Bad Säckingen, Germany, is ideal for anterior teeth.

Fourth Category: Polycrystalline solids

Solid sintered monophase ceramics, like Procera AllCeram Alumina, Nobel Biocare AB, Göteborg, Sweden, are dense, air-free, glass-free polycrystalline structures created by directly sintering crystals [78].

The fracture toughness of a material indicates its resistance to crack propagation. The physical properties of zirconia appear to make it appropriate for use in anterior and posterior fixed partial dentures with multiple units. Clinical studies regarding the material have not indicated any issues with the zirconia framework [79,80].

### 3.2. Manufactoring Techniques of Dental Ceramics

The manufacturing techniques are summarised in the table below (Table 3), along with the material’s ability to be etched prior to adhesive bonding, while taking the manufacturer’s instructions into consideration.

### 3.3. The Applicability and Clinical Considerations of Dental Ceramics

The significance of therapy now extends beyond just clinical and medical aspects. Modern patients anticipate that dental restorations will replicate the optical characteristics of their natural teeth, requiring the addition of an aesthetically pleasing solution. As metal-free restoration techniques were developed, efforts were made to find ways to speed the development of restorations as well as the duration of patient treatment. Consequently, the press method (moulded technology) was developed [81,82,83], which has gained popularity in dental laboratories for its capacity to create restorations of exceptional quality. Furthermore, CAD/CAM methodologies are already prevalent in dentistry [83,84]. Industrial systems engineering processing methods have been modified to meet dental needs, enabling the fabrication of prosthodontic dental restorations. Dental offices and labs have adopted CAD/CAM methods, producing novel ceramic materials like biomaterials for dental restorations. This technology has led to faster turnaround times, reduced costs, and improved patient outcomes. New ceramic materials have been developed for CAD/CAM systems, meeting unique requirements for dental restorations like strength, durability, and biocompatibility. CAD/CAM technology also allows for same-day restorations, eliminating the need for multiple appointments and temporary restorations. Overall, CAD/CAM technology has improved patient care and workflow, but the applicability (Table 4) of materials remains dependent on the clinician’s skills and knowledge.

The selection of ceramic and hybrid materials for dental restorations hinges on balancing mechanical properties, aesthetic demands, and the specific clinical indication. Feldspathic and leucite-reinforced ceramics excel in anterior aesthetics for veneers, inlays, and onlays due to their translucency, though they exhibit lower strength. Lithium disilicate offers a significant step up in strength, broadening its applicability to anterior and posterior crowns and short-span bridges while maintaining good aesthetics. Zirconia-reinforced lithium silicate aims to combine higher strength with aesthetic benefits, suitable for a wide range of restorations, including implant-supported crowns.

For high-stress applications like posterior multi-unit bridges and implant abutments, zirconia stands out due to its superior strength and fracture toughness. While traditional zirconia had lower aesthetic appeal, newer high-translucency formulations and layered techniques are improving its use in more visible areas. Hybrid ceramics offer a unique combination of elasticity and moderate strength, making them less abrasive and potentially more resilient, suitable for various dental restorations and implant crowns.

Modern dental restorative materials should be strong, durable, and mimic natural teeth’s appearance. They should be easy to handle, requiring no additional treatment post-CAD/CAM, and provide an instant, comfortable fit for patients. By satisfying colour, translucency, and surface texture requirements with minimal dental tissue waste, these materials ensure the longevity and functionality of the prosthodontic restoration [83]. The material chosen to create a long-lasting, aesthetically favourable restoration depends on the patient’s preoperative condition. Dental ceramics possess limitations as restorative materials primarily due to their inability to withstand all functional stresses. Consequently, their application in the lateral and posterior areas was initially limited; however, advancements in these materials [85] have enabled their use as posterior long-span fixed partial prosthetic restorations and frameworks on dental implants [86]. In comparison to other dental materials, such as metals, dental all-ceramics exhibit low fracture toughness [87]. Ceramics fused to metal systems still combine metal’s excellent mechanical characteristics with ceramics’ exceptional aesthetic qualities [14]. Certain patients may experience complications with specific metals utilised as restorative materials in dentistry. Allergies, gum discolouration [88], and the liberation of metallic ions into the gingival tissue are among the manifestations of these concerns [89]. These constraints have stimulated the research and development of metal-free, aesthetically superior ceramic systems.

To reconstruct anterior teeth, aesthetically pleasing glass ceramics with strength values ranging from 200 to 400 MPa are frequently selected [30]. Considering their poor strength, leucite glass ceramics are only suggested for use in the manufacturing of anterior crowns, inlays, and onlays. The introduction of lithium disilicate glass ceramics has considerably widened the indications for glass ceramics [90]. Due to the significant stresses generated in the posterior dental arch areas, glass ceramics are utilised exclusively for single-tooth restorations (inlays, onlays, and crowns). Consequently, high-strength and resilient oxide ceramics, such as ZrO_2_, are commonly used in the design of long-span bridges for lateral restorations. Glass ceramics are then either pressed to or built up on top of these oxide ceramic substructures to mimic the aesthetic and tribological properties of the natural dentine.

The most advanced generation of ceramic materials offers compelling alternatives regarding material selection and manufacturing techniques. Successful functioning of these restorations necessitates a deeper understanding of the material dynamics in relation to the restoration design and intended application.

### 3.4. Properties of Ceramic Materials

Dental ceramics have outstanding aesthetics, are chemically inert in the oral cavity, and are highly biocompatible with the soft tissues of the mouth. The structure of porcelain restorations is probably their most important mechanical feature. The material’s structure is influenced by the ceramic’s composition and surface integrity, and the presence of surface components also influences strength. The mechanical and optical properties of the materials are determined by the type, quantity, size, and thermal expansion coefficient of the crystalline phases. Dental ceramics have exceptional resistance to tensile and shear stresses, yet they demonstrate inadequate performance under compressive loads [23,25]. This imparts a brittle nature to ceramics, increasing their susceptibility to fracture under tensile stress [91]. Cracks originating from the contact area at the occlusal surface [91,92], from the cementation surface beneath the contact, and from the margins of crowns and connectors in multi-unit fixed partial dentures exemplify various clinical fracture mechanisms of ceramic structures [93,94]. Dental ceramic prostheses fail due to structural defects. Microcracks measuring less than one millimetre may develop during the fabrication of ceramic prostheses or due to masticatory forces in the oral cavity [12]. The durability and longevity of dental ceramic restorations are greatly affected by their fatigue strength. The cyclic application of loads and chemically accelerated, rate-dependent fracture progression in a humid environment can elucidate fatigue [95,96]. Early cracks are penetrated by water, which dissolves the cohesive bonds holding the crack walls together. This results in a gradual spreading of a crack, which progressively advances over time, accelerates under heightened stress levels, and ultimately fails [91].

Artificial or natural teeth may be damaged by the extremely hard surfaces of ceramics. Ceramics function as efficient thermal insulators, and their coefficient of thermal expansion is nearly equivalent to that of a natural tooth. The removal of voids during sintering results in the loss of any residual water and binders from the material during firing. This results in about 30–40% volume shrinkage. In order to handle this shrinkage during the manufacturing process of a porcelain restoration, meticulous control of the condensation and firing processes must be maintained [12,25,97].

There may be considerable compositional differences between categories of glass-based systems with a crystalline phase and the ceramic group with interpenetrating phase ceramics, as these material categories manifest alongside a diverse array of commercial products. The specific chemical structure may influence the ceramics’ visual aesthetics and mechanical characteristics in features like high fracture toughness, low abrasive characteristics, flexural strength, and wear resistance [42]. The sensitivity of ceramic materials to hydrofluoric acid may influence their capacity to form a solid bond between resin and ceramic during the etching process [14]. Etchable glass-based systems (including glass-based systems and glass-based systems with a crystalline phase) facilitate bonding, thereby enhancing the long-term success of prosthetic treatment. Materials with a crystalline structure, such as interpenetrating phase ceramics and polycrystalline solids, cannot be etched, consequently complicating the bonding process considerably. The dental ceramic categories of glass-based systems and interpenetrating phase ceramics can exist as a powder, which is later processed using a wet brush technique, or they may be formed into a block suitable for pressing or machining. The final restoration frequently exhibits a considerably greater quantity of bubbles and imperfections compared to pre-manufactured blocks, resulting in powder/liquid systems generally possessing much lower strength. As summarised in Table 5, the clinician should evaluate the material properties to obtain accurate applicability guidelines based on the practical indications of ceramics. The number of checkmarks indicates the level of favourable characteristics, with two checkmarks representing a standard level and three checkmarks indicating enhanced properties for the respective optical and bonding parameters.

Ceramics’ major limitations are brittleness, weak fracture toughness, and low tensile strength. Strategies adopted to minimise the disadvantages of ceramics include techniques for reinforcing brittle materials and methods for designing components that decrease stress concentration and tensile stress [25]. Two methods for strengthening brittle materials are the formation of residual compressive stress within the material’s surface and the inhibition of fracture propagation within the material. The surfaces of glass and ceramic materials undergo residual compressive stresses to enhance strength. The tensile strains generated during functioning are balanced by the applied compressive loads. Compressive stresses can be generated by any of the three processes: chemical tempering, thermal tempering, and thermal compatibility [12,25]. Glasses or ceramics are reinforced by incorporating a dispersed crystalline phase, which inhibits the propagation of cracks within the material. Feldspathic ceramics, known for their low mechanical strength, have been improved over time, which included up to 50% alumina and led to a ceramic with a flexural strength of 120–150 MPa, enabling a wider range of clinical applications [39]. These ceramics also have excellent optical properties, with a refractive index similar to enamel and dentin [98].

Synthetic glass ceramics have a microstructure composed of dispersed crystals encased in a translucent glassy phase or matrix. The crystalline phase enhances mechanical qualities [6], reducing fracture development and preventing crack propagation. It allows the ceramic material to adapt to enamel and dentin colours, provides stability during manufacturing, and resists occlusal stresses [43]. The mechanical characteristics of these ceramics are influenced by intrinsic and extrinsic factors, including crystal size, number, and distribution pattern. These ceramics are influenced by factors such as humidity, cyclic loading, thermical shocks, and pH levels [27,43].

Leucite-based ceramic is a type of dental material with a heterogeneous glass ceramic state. It has a crystalline content of 45% and is homogenously distributed within the glass matrix. Its high silica content enhances translucency, fluorescence, and opalescence [14]. Its flexural strength of 160 MPa is due to its crystalline composition, which absorbs fracture energy, preventing crack propagation [37]. Although it is double the strength of conventional feldspathic ceramics, it is not suitable for posterior bridges [42].

Lithium disilicate crystals have a platelet-like microstructure due to their partially crystallised “blue state” of lithium metasilicate, which has a flexural strength of 130 MPa, making them favourable for milling. Heat treatment through crystallisation controls the final microstructure, with lithium silicate ceramics having the highest strength among all silicate ceramics, with a flexural strength of around 407 MPa [42]. However, flourapatite-based glass ceramics have a lower flexural strength (90–110 MPa) compared to other all-ceramic materials used in prosthetic infrastructures and frameworks [6,99].

Zirconia-reinforced lithium silicate (ZLS) ceramics, while offering enhanced strength due to zirconia inclusion (370 MPa), require polishing, staining, and glazing for optimal aesthetics. Although glazing can contribute to surface strengthening, the primary mechanical improvement stems from the zirconia particles reinforcing the lithium silicate crystal and glass matrix. Potential thermal incompatibility between these phases can lead to residual stress and microcracking, impacting mechanical reproducibility [44]. Surface treatment, involving 4.9% HF gel for 20 s, showed the best microstructure retention. Nevertheless, increased etching periods and hydrofluoric acid concentration led to surface deterioration [98].

Concerning varieties of glass-infiltrated ceramics, the flexural strength and fracture toughness of alumina and zirconia (600 MPa) and that of mainly alumina (500 MPa) do not vary significantly. Furthermore, acid etching using HF acid has little effect on the surface microstructure of these ceramics.

To prevent the propagation of the fracture, two specific types of dispersions, namely alumina or partially stabilised zirconia, are used [12,27]. Alumina is a rigid, crystalline substance that can enhance glass by inhibiting fracture propagation. Under stress, stabilised zirconia can modify its crystal structure, thereby enhancing its strength [12].

Due to its distinct physical properties, zirconia is twice as resistant and strong as ceramics that contain alumina. The flexural strength values for this material range from approximately 900 to 1400 MPa [100]. Flexural strength and clinical performance are not directly correlated, which is significant. Fracture toughness is another notable physical property, reported to have range from 8 to 10 MPa m^1/2^ for zirconia [101]. The ratio of the glassy phase to the crystalline phase must be optimised to achieve the final ceramic restoration with adequate mechanical and optical properties. High strength requires an increased proportion of the crystalline phase, whereas superior translucency demands a higher percentage of the glassy phase. To equalise the two material phases, it is fundamental to reduce the intrinsic limitations of high abrasion resistance, fracture toughness, and tensile stress resistance. Numerous efforts have been undertaken to address these deficiencies, yet further action is required. A potential solution is to integrate additives that can improve the material’s fracture toughness and tensile strength while maintaining its abrasion resistance. An alternative method involves altering the material’s microstructure via heat treatment or alloying, which can enhance its mechanical properties [12].

Resin matrix ceramics are renowned for their elasticity, making them ideal for dental restorations due to their ability to distribute occlusal loads more evenly. They are highly flexible and rigid, making them suitable for bonded restorations [6] and the higher fatigue strength compared to glass ceramics describe them as ideal for unstable occlusal situations. Resin matrix ceramics are aesthetically pleasing due to their light-transmissive, light-scattering, and fluorescence properties [102]. Available in various translucencies and multicoloured blocks with remarkable machinability and low hardness, they allow for smoother restorations and greater precision in prosthetics. The manufacturing thicknesses are 0.5 to 0.3 mm, making them suitable for conservative restorations respecting residual tooth structure [103].

Moreover, modern manufacturing methods like 3D printing can be applied to create complex structures that evenly distribute stress and minimise the probability of fractures. A multidisciplinary strategy integrating materials science, engineering design, and advanced manufacturing technologies is ultimately demanded to achieve long-term satisfying clinical outcomes.

## 4. Adhesive Materials in All-Ceramic Dentistry

The selection of restorative materials and cements in therapeutic settings has become more complex due to new cement innovations. Dentists now have a wide range of choices to meet modern restoration standards but must also consider various clinical situations that may not work with certain prosthetic materials [7]. Since the ceramic surface treatment before cementation differs depending on the kind of ceramic used, the clinician must understand the ceramic type, surface treatment, cementation material, and process to reach a satisfactory conclusion [5]. Practitioners must carefully consider the properties and indications of each material, including bond durability, aesthetics, and biocompatibility, while also considering the patient’s specific requirements. Staying current with dental materials and treatment planning ensures patients receive the best possible care. The long-term success of restorations depends on the choice of the luting agent and cementation technique [8], as well as adequate tooth and restoration surface preparation. Complying with the manufacturer’s requirements and allowing the cement to set before exposing the restoration to biting stresses define the restoration’s success. Several luting agents, including glass ionomer cement and resin cement, which are the most typically utilised for ceramic restorations, have been widely studied in the literature [12,104].

Glass ionomer cements adhere effectively to dental tissue and release fluoride, assisting in the prevention of secondary caries. Conversely, resin cements have superior bond strength and enhanced aesthetics due to their ability to be colour- and shade-matched. An appropriate cementation procedure is required for the durability of a restoration. This demands proper isolation, decontaminating the tooth surface, and providing sufficient time for the cement to set [105].

The ceramic surface must be modified through a particular surface treatment protocol (Table 6) to assure proper adhesion with the luting agent.

Mechanical techniques include air abrasion/sand blasting, diamond stone burs, sandpaper discs, and laser therapy [9,10]. Conversely, excessive surface roughening must be avoided as it may lead to crack initiation and propagation within the ceramic, ultimately resulting in the fracture of the ceramic restoration. Chemical modifications to the ceramic surface can be achieved through etching to enhance the mechanical retention of the adhesive or by affecting the surface’s affinity for adhesive materials [12,13]. Studies reveal that chemical conditioning procedures like silanization improve the composite resin bond’s adherence to the ceramic [17]. The silanization process chemically bonds the silica in the dental ceramic with the acrylic group of the composite resin. To strengthen the bond between adhesive resins and ceramics, a combination of chemical and mechanical conditioning techniques is suggested [12,18].

Dental cement should have biocompatibility with pulp and soft tissues, low solubility, long working time, radiopacity, and optimum film thickness for prosthetic restorations. It should have high shear, tensile, and compressive strength and excellent adherence to the substrate and restorative material. It should be easy to mix and handle and excess cement should be eliminated easily after cementation. These properties ensure restorative treatment retention [21].

Adhesive cements provide superior strength and bonding properties compared to conventional cements, which rely on mechanical interlocking for retention. Consequently, they serve as an optimal alternative for low-strength restorations since they provide enhanced support and retention. Traditional cements include zinc oxide phosphate, carboxylate cements, glass ionomers, and resin-modified ionomer cements [21]. However, since adhesive cements have stronger bonding qualities, they are a preferable option. In order to create a strong, long-lasting bond that can sustain significant loads, they chemically adhere to the substrate and the repair surface. Furthermore, adhesive cements have a thinner layer compared to conventional cements, which reduces the risk of marginal leakage and hence improves aesthetics. While conventional cements are used in dentistry, adhesive cements provide a more stable and durable alternative for various restorative components [106].

The clinical success of all-ceramic restorations depends on the cementation process and cement selection due to the many components and properties. Bonding agents, adhesive systems, and resin cements have seen major advancements in dental medicine during the last few years. The bonding mechanisms of enamel and dentine are quite different. Cements have been developed to establish a connection with the organic component of dentin through the dentinal adhesion process. “Resin cements” or “composite resin cements” bond to the organic component of dentin by the dentin adhesion process. The development of a hybrid layer of dentine collagen fibres is the foundation of these adhesion systems. All glass ceramic restorations will require hydrofluoric acid demineralisation prior to the application of a silane-based primer, bonding agent, and resin cement [7]. Since high-strength ceramic restorations based on zirconium oxide or aluminium oxide infrastructure cannot be bonded as they are not acid-etched, a surface treatment including sandblasting, silane application, and certain primers is necessary to cement the restoration [45]. No dental cement is available that fulfils all biomechanical requirements completely. The cementation of diverse all-ceramic restorations is no longer traditional due to the emergence of novel materials and procedures. The clinician will choose the most suitable cement based on the advantages and disadvantages of each, according to the clinical situation, and will adapt each adhesive strategy for particular clinical considerations (Table 7).

Total-etch resin cement systems etch enamel and dentine using a 30–40% phosphoric acid etching process. The smear layer is eliminated, and the dentin canaliculi are exposed during this demineralisation procedure. Following demineralisation, the adhesive is applied to the preparation to facilitate the adhesion of the cement to the tooth. These cement types, together with their corresponding adhesives, are either light-cured or dual-cured. Total-etch resin cements significantly improved the adhesion strength of resin-based cements and reduced microinfiltrations [107]. This group has the superior cement–tooth bond but requires many procedures to adhere the ceramic, composite resin, or metal to the tooth. This multi-step method may compromise the effectiveness of the bonding since each stage presents a possible source of errors.

Self-etch systems condition the tooth surface using a self-etch primer prior to the application of the mixed cement over the primer. These cements establish bonds with dental structures that are almost comparable to those in the total-etch cement group. Due to their ease of use, self-etch cements are favoured by dentists. These cements displayed inferior bonding strength to enamel compared to total-etch methods. Self-adhesive primers streamline clinical procedures, improve handling efficiency, reduce the risk of operational errors, and increase procedural sensitivity [108].

Self-adhesive cements are monocomponent products that have strong adherence to dentin, enamel, and ceramic without requiring supplementary bonding agents. These cements may bond to an untreated tooth surface that has not undergone pretreatment, etching, priming, or the application of an adhesion agent, facilitating a one-step cementation process [109]. Phosphoric acid methacrylate is incorporated into the resin of these cements, establishing a chemical bond with the filling material particles and dentin in the presence of water [110]. The use of both etching and adhesion compounds enhances enamel adherence. The bond strength of self-adhesive resin cements may be increased by selective demineralisation of enamel and/or dentin surfaces and indirect restorations. Adhesion may be diminished by etching the dentine with phosphoric acid and using a bonding agent before the application of the cement [21].

Self-adhesive resin cements have lower adhesive performance and bond strengths to enamel and dentin compared to conventional multi-step cements [111], making them unsuitable for restorations with minimal retention and resistance forms, such as resin-bonded bridges. They also have weaker enamel bonding and a higher tendency to discolour [112], making them unsuitable for veneers. A five-year prospective clinical study [113] found that total-etch resin cement had better marginal continuation and adaptation. Table 8 briefly describes resin cement’s properties and clinical indications.

The optimal polymerisation of light-activated resins requires the light source to be close to the tooth, regardless of the photo-initiator used. The light-curing unit’s tip angle should be kept at a 90-degree angle to optimise light transmission to the material’s depth [116].

Chemically activated cements have applications for thick restorations and bonding posts and crowns made of opaque materials like ceramics or metallic copings, which are ideal for long-term properties in areas unreachable by light. Even so, their working time is limited compared to their extended setting time, and they tend to become yellowish due to high activator concentration. Refrigeration can extend their shelf life, but the main challenge remains the uniform mixing of pastes for even setting [117].

Dual-cured resin cements aim to combine photo- and chemically activated systems for optimal conversion in deep restoration areas [118]. Nonetheless, inadequate photo-activation can lead to reduced conversion and hardness [119], increased solubility, lower flexural and compressive strengths, and inferior bond strength to dentin compared to directly light-cured dual cements. Improper photoactivation can result in poorer results [119,120].

Researchers are exploring the use of restorative composite resin as an alternative luting material due to its advantages over traditional resin cements, such as higher strength, cost-effectiveness, lower marginal deterioration, and a wider range of colour choices (Table 9) [121].

In spite of that, composite resin contains a higher proportion of filler particles, making it more viscous and less flowable [122]. To decrease film thickness and viscosity, methods like preheating and ultrasonic vibration have been explored. However, some composite resins have not achieved a film thickness of <50 μm, which can affect fracture resistance [117] and marginal adaptation of restorations [121]. A new class of resin cements, universal resin cement, which can be used in conventional, selective etch, or self-adhesive modes [118], simplifying the clinical decision to select the appropriate cementation technique for each case.

## 5. Limitations

This literature review, while aiming to provide a comprehensive overview of all-ceramic materials in modern digital dentistry, has several limitations. Firstly, the electronic search was conducted by focusing on publications within the 2008 to 2025 timeframe. This selection was made to reflect contemporary advancements relevant to current clinical practice; however, it may have excluded valuable evidence published prior to 2008. It is important to note, however, that foundational articles and key research published before 2008, which provide essential context and complete the previous perspective of this topic, are referenced within this review. Secondly, the inclusion criteria were restricted to articles published in the English language. This decision, based on the linguistic expertise of the authors, may have resulted in the omission of relevant research findings published in other languages. Readers should consider these limitations when interpreting the findings and conclusions of this review.

## 6. Conclusions

Modern dentistry has experienced a transformative evolution with the integration of CAD/CAM technology, significantly impacting the selection and application of all-ceramic materials. This manuscript highlights the essential shift from traditional to digital procedures for clinicians aiming to enhance patient outcomes. The study emphasises the necessity of choosing materials that provide exceptional strength and durability while also considering aesthetic factors, including colour stability and translucency, essential for achieving aesthetics. CAD/CAM technology enables clinicians to maintain precise control over design and fabrication, thereby optimising appointments, improving patient comfort, and promoting interdisciplinary collaboration. This guide serves as a practical resource for clinicians managing the complexities of all-ceramic material selection in contemporary digital dentistry, assisting them to achieve reliable and long-term restorative treatments.

In practical application, the selection of prosthetic materials should be guided by a comprehensive assessment of the clinical scenario, encompassing the biomechanical demands of the restoration, the aesthetic expectations of the patient, and the long-term stability of the chosen material and luting system. For anterior restorations, highly translucent ceramics like feldspathic porcelain and lithium disilicate are preferred for their optical properties, while acknowledging their need for adequate support and careful occlusal management. In posterior regions or for load-bearing restorations, high-strength materials such as zirconia and zirconia-reinforced lithium silicate offer enhanced durability and fracture resistance, with advancements in translucency expanding their aesthetic applications. Hybrid ceramics present a versatile option, balancing flexibility and moderate strength for various indications. The long-term success of these restorations is heavily reliant on the appropriate selection and application of resin cements, with factors like bond strength, polymerisation mechanism, and material compatibility being decisive for predictable outcomes.

Within the limitations of this study, its findings indicate the importance of continuous professional education, focusing on the need for comprehensive guidance on the physical, mechanical, biological, and aesthetic properties of commonly used materials to encourage informed decision-making in accordance with modern advancements. Further research is necessary to assess appropriate workflows for adhesive systems, surface preparation, and conditioning methods for ceramics, including long-term clinical trial follow-ups for in vivo performance, intaglio surface treatment protocols, novel resin cement materials, and the predictability and durability of minimally invasive all-ceramic restorations using fully digital workflows.

In conclusion, an accurate understanding of material characteristics along with appropriate adhesive cementation procedures is essential for the effective integration of all-ceramic restorations in modern digital dentistry. This review focuses on the importance of research-based choices in the attainment of predictable aesthetics, enhanced resistance, and long-term clinical success in modern restorative dentistry by offering clinicians a clear guide to material selection and cementation protocols.

## Figures and Tables

**Table 1:** Chemical structure of dental ceramics [25].

Components	Functions
Feldspar	The lowest fusing component melts and flows first during firing, enabling these components to solidify.
Silica	Strengthens the prosthodontic ceramic restoration and maintains its original form at the temperature typically used to fire porcelain, adding stability to the mass during heating by acting as a framework for the other components.
Kaolin	It is used as a bond; at the same time, it acts on unfired porcelain by making it more mouldable and gives opacity to finished porcelain.
Glass modifiers	They function as flux and compromise the silica network’s integrity.
Colour pigments	To create the restoration, the suitable shade.
Zr/Ce/Sn oxides and uranium oxide	To generate the right amount of opacity.

**Table 2:** Compositions and properties of glass-infiltrated ceramics.

	Composition	Strength	Translucency
VITA In-Ceram™ SPINELL	Alumina and magnesia	400 MPa	High translucency
VITA In-Ceram™ ALUMINA	80%, alumina	500 MPa	Optimal translucency
VITA In-Ceram™ ZIRCONIA	Alumina and zirconia	600 MPa	High opacity

**Table 3:** The manufacturing methods and the etching capacity for adhesive bonding.

Material	Fabrication Method	Etchable
**Glass matrix ceramics**		
Felspathic ceramics	Refractory die, platinum foil, press	YES
**Synthetic ceramics**		
Leucite-based	Press or CAD/CAM	YES
Lithium disilicate and derivatives	Press or CAD/CAM	YES
Fluorapatite-based	Press or layering	YES
**Glass-infiltrated**		
Alumina	CAD/CAM or Slipcasting	YES
Alumina and magnesium	CAD/CAM or Slipcasting	YES
Alumina and zirconia	CAD/CAM or Slipcasting	YES
**Polycrystalline ceramics**		
Alumina	CAD/CAM	NO
Stabilised zirconia	CAD/CAM	NO
Zirconia-toughened alumina and alumina-toughened zirconia	CAD/CAM	NO
**Resin matrix ceramics**		
Resin nanoceramics	CAD/CAM	NO
Glass ceramics in a resin interpenetrating polymer network	CAD/CAM	YES
Zirconia–silica in a resin interpenetrating polymer network	CAD/CAM	NO

**Table 4:** Clinical applicability of dental ceramics.

Ceramic	Manufacturer	Clinical Indications
Feldspar	VITABLOCS, VITA Zahnfabrik: Mark I (1985); Mark II (1991); VITA Triluxe (2003); VITA Trilux forte (2007); VITA Real Life (2010).	Veneers, inlays, onlays, partial crowns, crowns (anterior and posterior area), veneering CAD/CAM material for multi-unit fixed partial restoration substructure made from oxide ceramic
Leucite	IPS Empress CAD, Ivoclar Vivadent (2006); IPS Empress CAD Multi	Veneers, inlays, onlays, partial crowns, and crowns (anterior and posterior area)
Lithium disilicate	IPS e.max CAD, Ivoclar Vivadent (2006)	Veneers, inlays, onlays, partial crowns, crowns (anterior and posterior area), hybrid abutments and crowns, 3-unit fixed partial dentures (anterior and premolar area), veneering CAD/CAM material for multi-unit fixed partial restoration substructure made from IPS e.max ZirCAD
Zirconia-lithium silicate	Celtra Duo, Dentsply (2013); VITA Suprinity VITA Zahnfabrik (2013)	Veneers, inlays, onlays, partial crowns, crowns (anterior and posterior area), implant-supported crown
Zirconia	Vita In-Ceram YZ VITA Zahnfabrik (2002); IPS e.max ZirCAD Ivoclar Vivadent (2006)	Crowns and multiple-unit fixed partial dentures (anterior and posterior area, curved and long-span), cantilever bridges, implant abutments, adhesive anterior bridges, primary telescope crowns, inlay bridge framework
All zirconia	Lava Plus High Translucency Zirconia 3M ESPE (2012); Cercon True Color, Dentsply, Degudent (2015); Zenostar Full Contour Zirconia Wieland Dental lvoclar Vivadent (2013).	Crowns and multiple-unit fixed partial dentures (anterior and posterior area, curved and long-span), inlay bridge framework, primary telescope crowns, cantilever bridges, implant abutments, and adhesive anterior bridges
Hybrid	Lava Ultimate CAD/CAM Restorative, 3M ESPE (2011); VITA Enamic, VITA Zahnfabrik (2013); Vita Enamic multiColor (2017); CERASMART GC (2014).	Veneers, inlays, onlays, partial crowns, crowns (anterior and posterior area), implant-supported crowns

**Table 5:** Characteristics of feldspathic and synthetic glass matrix ceramics.

Material	Mechanical Properties			Optical Properties	Bonding
	Bond Strength Resistance (MPa)	Toughness (MPa/m^2^)	Elesticity (GPa)		
Feldspathic ceramics	60–70	1.26	70	✓✓✓	✓✓✓
Lithium disilicate	360	2.25–2.75	95–102	✓✓	✓✓✓
Leucite-based	160	1.3	62–70	✓✓	✓✓✓
Fluorapatite-based	90	0.7–1	60–80	✓✓✓	✓✓✓
Zirconia-reinforced lithium silicate	370–420	2.6	70–108	✓✓	✓✓✓

**Table 6:** Adhesive cementation methods by ceramic type.

Ceramic Composition	Filler	Ceramic Surface Treatment Protocol
Predominantly Glass Ceramic	Aluminium oxide	10% HF for 1 min, rinse and dry, silane for 1 min, air dry
Particle-Filled Glass Ceramic	Leucite	5% HF for 1 min, rinse and dry, silane for 1 min, air dry
	Lithium disilicate	5% HF for 20 s, rinse and dry, silane for 1 min, air dry
	Glass-infiltrated alumina	Air abrasion with tribochemical silica coating/aluminium oxide, 10MDP agent, air dry
Polycrystalline Ceramic	Aluminium oxide	Air abrasion with aluminium oxide, 10MDP agent, air dry
	Zirconium oxide	Air abrasion with 50-micrometre aluminium powder at 7 pounds per square inch, 10MDP agent, air dry

**Table 7:** Classification of resin cements according to adhesive protocol.

Resin Cement	Clinical Protocol	Materials Characteristics
Total-etch	Demineralization with 30–40% phosphoric acid, followed by adhesive application	Excellent cement-to-tooth bond strength Reduced microleakage Long-term predictability Multiple-steps technique
Self-etch	Self-demineralizing primer, followed by pre-mixed cement application	User-friendly Good bond strength Less risks of error
Self-adhesive	A single component, phosphoric acid is included in the resin	Capable of bonding to the surface of an untreated tooth “Selective demineralization” can be incorporated to boost bond strength

**Table 8:** The polymerisation mechanism classification of resin cement [21,114,115].

Resin Cement	Polymerization Mechanism	Characteristics	Indications
Photopolymerizable	Light-activated photo-initiators	Chromatic stability Increased working time	Thin-layered ceramic restorations Restorations without metallic core Aesthetic restorations
Self-polymerizable	Chemical reaction	Applicability in unreachable areas by photopolymerization	Endodontic post and core Restorations with metallic core Metallic restorations Ceramic restorations thicker than >3 mm
Dual dental cement	Light-activated photo-initiators and chemical reaction	User-friendly High aesthetic results Very good adhesive strength Rapid seal of the marginal fitting area	Thick ceramic restorations Opaque ceramic restorations Suitable for any type of all-ceramic restoration

**Table 9:** Colour considerations in adhesive ceramic cementation.

Ceramic Material	Translucency and Opacity Characteristics	Cement Shade Considerations	Colour Management Strategies
Feldspathic Ceramics	High translucency, closely to the optical properties of natural enamel	Highly influenced by underlying tooth structure and cement shade. Neutral/slightly lighter shades are often preferred unless masking is needed	Tooth substrate shade modification with opaquer may be necessary if underlying discoloration is present. Thin veneers require precise cement shade selection
Leucite-Reinforced Ceramics	Moderate translucency, slightly more opaque than feldspathic	Cement shade still plays a significant role	May require opaquer in cases of significant underlying discoloration
Lithium Disilicate	Varies in translucency, from high translucency CAD/CAM blocks to high opacity blocks	Shade selection depends heavily on the block translucency. High translucency requires careful cement matching. Medium/high opacity offer more masking potential but can still be influenced by cement	For high translucency blocks, consider the underlying colour of the prepared tooth shade carefully, as it can significantly affect the final shade
Zirconia (Monolithic)	Opaquer than glass ceramics. Newer generations can have higher translucency	Cement shade has less of a dramatic effect due to higher opacity but can still influence the final value and subtle colour nuances, especially with more translucent zirconia. Consider shades that complement the ceramic	Surface staining of the zirconia can be used to achieve desired shade effects. Opaque cements can be used without significant risk of negatively impacting the aesthetic outcome in most cases
Zirconia (Veneered)	The veneering porcelain’s translucency dictates the final aesthetic	The cement primarily affects the underlying zirconia coping’s influence on the veneer’s colour. Opaque cements block out the zirconia substructure and allow the veneer’s shade to dominate. Cement at the margins can still be visible and should be considered	Opaque cements are often preferred for the coping. The veneering ceramics shade and thickness are the primary determinants of the final visible colour
Alumina	High opacity	Cement shade has the least impact. The material masks the underlying tooth colour, but at the restoration margins, where if visible, the resin cement can impact the final appearance	Surface staining can be used for final shade adjustments

## Data Availability

The data analysed in this study may be requested from the corresponding author.
